# Does kin discrimination promote cooperation?

**DOI:** 10.1098/rsbl.2019.0742

**Published:** 2020-03-18

**Authors:** Gonçalo S. Faria, Andy Gardner

**Affiliations:** 1 School of Biology, University of St Andrews, Dyers Brae, St Andrews KY16 9TH, UK; 2 Institute for Advanced Study in Toulouse, 21 Allée de Brienne, Toulouse, France

**Keywords:** altruism, inclusive fitness, Jensen's inequality, kin selection, kin recognition, veil of ignorance

## Abstract

Genetic relatedness is a key driver of the evolution of cooperation. One mechanism that may ensure social partners are genetically related is kin discrimination, in which individuals are able to distinguish kin from non-kin and adjust their behaviour accordingly. However, the impact of kin discrimination upon the overall level of cooperation remains obscure. Specifically, while kin discrimination allows an individual to help more-related social partners over less-related social partners, it is unclear whether and how the population average level of cooperation that is evolutionarily favoured should differ under kin discrimination versus indiscriminate social behaviour. Here, we perform a general mathematical analysis in order to assess whether, when and in which direction kin discrimination changes the average level of cooperation in an evolving population. We find that kin discrimination may increase, decrease or leave unchanged the average level of cooperation, depending upon whether the optimal level of cooperation is a convex, concave or linear function of genetic relatedness. We develop an extension of the classic ‘tragedy of the commons' model of cooperation in order to provide an illustration of these results. Our analysis provides a method to guide future research on the evolutionary consequences of kin discrimination.

## 1. Introduction

Genetic relatedness is a key concept in the theory of kin selection [1–3]. In order for cooperation—or, indeed, any trait—to be favoured by kin selection, individuals must be able to interact with genetically related social partners. Three basic mechanisms may lead to social partners being genetically related [4]: (i) population viscosity, in which limited dispersal of individuals during their lifetimes ensures that even indiscriminate social interaction among neighbours is likely to be occurring between kin [1,2]; (ii) kin discrimination, by which individuals distinguish their genealogical relatives from unrelated individuals using genetic or environmental cues [1,2]; and (iii) the greenbeard effect, whereby genes can identify the presence of copies of themselves in other individuals, ascertaining genetic relatedness at this locus directly and irrespective of genealogical relationship [2,5,6]. Accordingly, kin discrimination is the key to classic nepotistic behaviour, in which individuals give preferential treatment to their genealogically close kin.

However, it is not clear whether and in what way kin discrimination should affect the overall level of cooperation within a population. Specifically, while kin discrimination would allow an individual to help more-related social partners over less-related social partners, it is unclear how the population average level of cooperation that is evolutionarily favoured should differ under kin discrimination versus indiscriminate social behaviour. Insofar as this issue has been raised within the kin-selection literature, the conventional view appears to be that individuals operating under a ‘veil of ignorance’ [7,8] with regard to their genetic relatedness to their social partners will tend to be favoured to behave more cooperatively than they would do were they able to behave nepotistically [9–11]. However, no formal analysis of this general problem has previously been undertaken, so it remains unclear whether this view is accurate and exactly what would drive this cooperation-promoting effect of ignorance.

Here, we perform a general analysis of the evolution of cooperation in order to assess whether, in what way and why the population average level of cooperation is modulated by the ability to behave nepotistically versus indiscriminately with regards to social behaviour. We then develop an extension of the classic ‘tragedy of the commons' model of the evolution of cooperation [12,13], which mathematically captures the tension between individual selfishness and group success, to illustrate the results of our general analysis in more concrete terms. We end by discussing the implications of our results for future theoretical and empirical research, focusing on the utility of our method in analysing the evolutionary consequences of lifting the veil of ignorance in a social population.

## 2. General analysis

We assume an infinite population separated into discrete groups within which individuals engage in public-goods cooperation. Let individual fitness be given by *w* = *W*(*x*, *y*, *z*), where *x* is the individual's own genetic ‘breeding’ value for investment into cooperation, *y* is the average genetic value across this individual's social partners and *z* is the average genetic value across the whole population. An increase in the average level of cooperation is favoured by natural selection when (d*w*/d*x*)|*_x_*_=*y*=*z*_ > 0, i.e. when Hamilton's rule −*c*(*z*) + *b*(*z*)*r* > 0 is satisfied, where −*c*(*z*) = (∂*w*/∂*x*)|*_x_*_=*y*=*z*_, *b*(*z*) = (∂*w*/∂*y*)|*_x_*_=*y*=*z*_ and *r* = d*y*/d*x* is the kin-selection coefficient of relatedness [1,2,14,15]. Accordingly, assuming that an intermediate evolutionarily stable strategy [16] exists, this ‘optimal’ level of investment into cooperation z* = *Z*(*r*) satisfies −*c*(z*) + *b*(z*)*r* = 0, as this is a necessary condition for evolutionary stability [17,18]. Note that while the marginal fitness effect −*c*(*z*) + *b*(*z*)*r* of an increase in cooperation is a linear function of relatedness *r*, the optimal level of investment z* need not be.

This optimum pertains to situations in which public-goods cooperation is governed by a single genetic trait, i.e. when there is only one context within which cooperation is performed, or if individuals are unable to adjust their level of cooperation according to context. If instead cooperation is performed in multiple contexts, and individuals are able to adjust their level of cooperation according to the optimum for each context separately, then a separate genetic trait must be considered for each of these different contexts. An example of this is when there is variation in relatedness between social groups and individuals are able to judge how related they are to their social partners and respond to this conditional information by adjusting their level of cooperation. Here, the condition for natural selection to favour an increase in cooperation in relatedness context *i* is −*c*(*z*) + *b*(*z*)*r*_i_ > 0, where *r*_i_ is the genetic relatedness pertaining to this particular context, and the optimal level of cooperation for this context is given by *z*_i_* = *Z*(*r*_i_) which satisfies −*c*(*z*) + *b*(*z*)*r*_i_ = 0.

In this case, the average level of cooperation across the population is given by ${\bar{z}}^{*}=\sum_{i}p_{i}z_{i}^{*}=\sum_{i}p_{i}Z(r_{i})$, where *p*_i_ denotes the proportion of individuals experiencing relatedness *r*_i_. It follows that, if *Z* is a linear function of its argument, then ∑_i_*p*_i_*Z*(*r*_i_) = *Z*(∑_i_*p*_i_*r*_i_) = *Z*(*r*), and hence ${\bar{z}}^{*}=z^{*}$, i.e. in this linear case the average level of cooperation across the population is exactly the same irrespective of whether individuals adjust their level of cooperation in light of kin discrimination versus expressing the same level of cooperation in all kinship contexts. By contrast, if *Z* is a nonlinear function of its argument, then the average level of cooperation across the population may depend on whether individuals respond facultatively to kinship information. In particular: if *Z* is a convex function of its argument, then, from Jensen's [19] inequality, we have ∑_i_*p*_i_*Z*(*r*_i_) ≥ *Z*(∑_i_*p*_i_*r*_i_), such that ${\bar{z}}^{*}\geq z^{*}$, i.e. the population average level of cooperation may be larger when individuals recognize and respond to kinship versus behave indiscriminately; and if *Z* is a concave function of its argument, then ∑_i_*p*_i_*Z*(*r*_i_) ≤ *Z*(∑_i_*p*_i_*r*_i_), such that ${\bar{z}}^{*}\leq z^{*}$, i.e. the population average level of cooperation may be lower when individuals recognize and respond to kinship versus behave indiscriminately.

## 3. An illustration

We now provide a concrete illustration of the general predictions made above using a development of the classic ‘tragedy of the commons' model of the evolution of cooperation [12,13]. We continue to consider an infinite population subdivided into social groups, with each group having the same amount of resource at its disposal. We now assume that in a proportion *q* of social groups the average relatedness of the constituent individuals is *r*_low_, and in a proportion 1 − *q* of social groups the average relatedness is *r*_high_. Without any loss of generality, we assume *r*_low_ ≤ *r*_high_, such that we may refer to the first type of group as low-relatedness groups and the second type of group as high-relatedness groups. We denote an individual's level of cooperation if finding herself in a low-relatedness group as *x*_low_ and her level of cooperation if finding herself in a high-relatedness group as *x*_high_, and we denote the overall level of cooperation in her group as *y*_low_ in the low-relatedness setting and as *y*_high_ in the high-relatedness setting. We assume that the overall fitness of the social group is proportional to the level of cooperation exhibited by its constituent members, and that individuals who behave relatively less cooperatively enjoy a relatively greater share of their group's fitness, such that the focal individual's fitness is given by

3.1 $$w=q{\left(\frac{1-x_{\mathrm{low}}}{1-y_{\mathrm{low}}}\right)}^{\beta }y_{\mathrm{low}}+(1-q){\left(\frac{1-x_{\mathrm{high}}}{1-y_{\mathrm{high}}}\right)}^{\beta }y_{\mathrm{high}},$$

where β≥0 describes the rate of decline in group share of fitness associated with an increase in relative cooperativeness.

In the absence of kin discrimination—i.e. under the constraint that *x*_low_ = *x*_high_ and *y*_low_ = *y*_high_—we find that the optimal level of cooperation is given by

3.2 $$z^{*}=\frac{r}{r+\beta (1-r)},$$

where *r* = *q r*_low_ + (1 − *q*) *r*_high_ is the average within-group relatedness across the population (see electronic supplementary material for details). Note that this optimum level of cooperation is of the form z* = *Z*(*r*), as derived in the previous section, and moreover, it is a concave function of relatedness when 0 < *β* < 1, a linear function of relatedness when *β* = 1 and a convex function of relatedness when *β* > 1. Also note that this exactly recovers Frank's [12,13] result for the special case where *β* = 1: here, the optimal level of cooperation is z* = *r*, and hence the optimal level of selfishness is 1 − *r*.

If instead individuals are able to adjust their level of cooperation according to the level of relatedness that they detect within their group—i.e. so it is possible that *x*_low_ ≠ *x*_high_ and *y*_low_ ≠ *y*_high_—then we find that the optimal level of cooperation is given by

3.3 $$z_{\mathrm{low}}^{*}=\frac{r_{\mathrm{low}}}{r_{\mathrm{low}}+\beta (1-r_{\mathrm{low}})},$$

for an individual in a low-relatedness group, and by

3.4 $$z_{\mathrm{high}}^{*}=\frac{r_{\mathrm{high}}}{r_{\mathrm{high}}+\beta (1-r_{\mathrm{high}})},$$

for an individual in a high-relatedness group (see electronic supplementary material for details), and this means that the overall level of cooperation in the population is given by

3.5 $${\bar{z}}^{*}=q\frac{r_{\mathrm{low}}}{r_{\mathrm{low}}+\beta (1-r_{\mathrm{low}})}+(1-q)\frac{r_{\mathrm{high}}}{r_{\mathrm{high}}+\beta (1-r_{\mathrm{high}})}.$$

**Figure 1.**
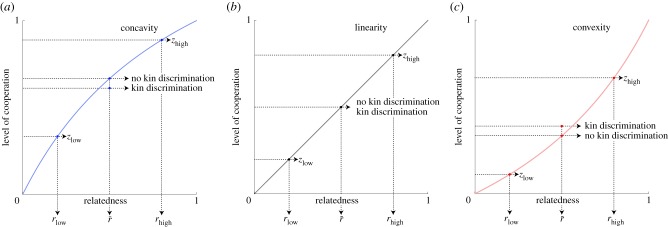
Comparison of a tragedy of the commons model when kin discrimination is absent versus when kin discrimination is present. When the candidate uninvadable investment of cooperation is a concave function of genetic relatedness (*a*), presence of kin discrimination leads to cooperation average being lower in the population than when kin discrimination is absent. When the candidate uninvadable investment of cooperation is a linear function of genetic relatedness (*b*), presence or absence of kin discrimination leads to the same cooperation average in the population. Finally, when the candidate uninvadable investment of cooperation is a convex function of genetic relatedness (*c*), presence of kin discrimination leads to cooperation average being higher in the population than when kin discrimination is absent. The following parameter values were used: frequency of different types of patches *q* = 0.5; relatedness *r*_low_ = 0.2; relatedness *r*_high_ = 0.8. Additionally, in (*a*) we assume *β* = 0.5, in (*b*) we assume *β* = 1, and in (*c*) we assume *β* = 2.

Comparison of the overall level of cooperation in the presence (equation (3.5)) and absence (equation (3.2)) of kin discrimination recovers the results of our general analysis: (i) kin discrimination decreases the overall level of cooperation (${\bar{z}}^{*}<z^{*}$) when cooperation is a concave function of relatedness (0 < *β* < 1; figure 1*a*); (ii) kin discrimination does not affect the overall level of cooperation (${\bar{z}}^{*}=z^{*}$) when cooperation is a linear function of relatedness (*β* = 1; figure 1*b*); and (iii) kin discrimination increases the overall level of cooperation (${\bar{z}}^{*}>z^{*}$) when cooperation is a convex function of relatedness (*β* > 1; figure 1*c*).

## 4. Discussion

In the social-evolution literature, there has been a tendency to associate ‘veil-of-ignorance’ scenarios with increased cooperation, such that individuals behave more cooperatively—on average—when they have less information [9–11]. Here, we have shown that ignorance concerning the relatedness of one's social partners may increase, decrease or leave unaffected the overall level of cooperation that is favoured in an evolving population, depending on whether the optimal level of cooperation is a convex, concave or linear function of relatedness. We have demonstrated this result both in general terms and also in the context of an illustrative, ‘tragedy-of-the-commons' setting.

Queller & Strassmann's [11] discussion of the role of ignorance in the evolution of cooperation is the only treatment, to our knowledge, that has provided a general prediction as to how ignorance of relatedness will modulate the overall level of cooperation. Generalizing across particular contexts—including conflicts in relation to meiotic drive, parent-of-origin-specific gene expression and queen–worker conflicts in social-insect colonies—they suggest that ignorance with respect to relatedness is a general promoter of cooperation (and their analysis also considered ignorance with respect to other factors, such as fitness pay-offs). Our mathematical analysis agrees that this will be the case provided that the optimal level of cooperation is generally a concave (i.e. decelerating) function of relatedness, but suggests that the opposite result will obtain in scenarios where cooperation is a convex (i.e. accelerating) function of relatedness. Our analysis provides no basis for believing that cooperation should be a concave as opposed to the convex function of relatedness and so, if this does happen to be the case across the majority of empirical scenarios, further theoretical and empirical investigation will be required in order to explain why this should be so.

In order to facilitate comparison between scenarios in which kin discrimination is present versus absent, we have assumed that the underlying relatedness structure of a population is independent of this aspect of a species' biology. However, it is feasible that any impact that kin discrimination has on the overall level of cooperation could itself modulate the incidence (*p*_i_) and corresponding relatedness coefficient (*r*_i_) for each of the situations in which individuals may find themselves, and such changes could further contribute to differences in the overall level of cooperation that is evolutionarily favoured. Depending on the details of a species’ biology, this complicating effect could either increase or decrease heterogeneity in the relatedness structure of the population, so it is difficult to make general predictions as to the difference this would make.

Similarly, we have assumed that the cost and benefit functions of cooperation (*c* and *b*) are also unchanged, for the sake of comparison, though in principle these could be modulated by kin discrimination in a number of ways. Finally, we have assumed that the personal-fitness effects of a given amount of cooperation for the actor and recipient are independent of their relatedness to each other; dependences may arise if, for example, failure to cooperate is more likely to lead to reprisals when social partners are less-related [20,21]. In the absence of reasons for suspecting that such complicating factors will always modulate cooperation in a particular direction, our main conclusion that there is no formal basis for supposing that there is a clear directional link between kin discrimination and the overall level of cooperation remains unchanged.

Kin discrimination occurs in relation to a variety of social-evolutionary scenarios, including sexual conflict [22], cooperation [23], sex allocation [24] and resource competition [25]. To our knowledge, no empirical study has investigated how kin discrimination affects the overall level of social behaviour, and it is our hope that our analysis will stimulate such work in the future. Note that this issue is distinct from the question of whether and when kin discrimination itself will be evolutionarily favoured. When inference of relatedness is possible from environmental or genetical cues, individuals are expected to kin discriminate provided the cognitive (and other) costs are not too large and there is sufficient variance in relatedness for the cues to be informative [26]. However, genetic kin discrimination is only expected to occur in limited circumstances where there is strong balancing selection acting to maintain allelic variation at those loci used in the identification of kin, as otherwise the benefits of being recognized as kin would lead to common alleles increasing in frequency and rare alleles being lost, such that these become poor indicators of kinship [27–29].

## Supplementary Material

Electronic Supplementary Material

## Data accessibility

This article does not contain any additional data.
