# Supplementary material for: Does kin discrimination promote cooperation?
Source: Biol Lett. 2020 Mar 18;16(3):20190742. doi: 10.1098/rsbl.2019.0742 (PMC7115181; doi:10.1098/rsbl.2019.0742)
Supplement: Electronic Supplementary Material [file rsbl20190742supp1.docx]

**Electronic supplementary material of the paper “Does kin discrimination promote cooperation?” by Faria GS & Gardner A**

The relative fitness of the focal individual in the context of the illustrative model of the main text is:

$W= c_{\mathrm{low}}\frac{w_{\mathrm{low}}}{\bar{w}_{\mathrm{low}}}+c_{\mathrm{high}}\frac{w_{\mathrm{high}}}{\bar{w}_{\mathrm{high}}}$, (A1)

where: *w*_low_ is the absolute fitness of an individual experiencing relatedness *r*_low_; $\bar{w}_{\mathrm{low}}$ is the average absolute fitness of an individual experiencing relatedness *r*_low_; *w*_high_ is the absolute fitness of an individual experiencing relatedness *r*_high_; $\bar{w}_{\mathrm{high}}$ is the average absolute fitness of an individual experiencing relatedness *r*_high_; *c*_low_ is the reproductive value of an individual experiencing relatedness *r*_low_, which in this case is

$c_{\mathrm{low}}=\frac{q \bar{w}_{\mathrm{low}}}{q \bar{w}_{\mathrm{low}}+(1-q)\bar{w}_{\mathrm{high}}}$; (A2)

*c*_high_ is the reproductive value of an individual experiencing relatedness *r*_high_, which in this case is

$c_{\mathrm{high}}=\frac{(1-q) \bar{w}_{\mathrm{high}}}{q \bar{w}_{\mathrm{low}}+(1-q)\bar{w}_{\mathrm{high}}}$; (A3)

*q* is the proportion of patches in the population experiencing relatedness *r*_low_; and 1 – *q* is the proportion of patches in the population experiencing relatedness *r*_high_. Note that $\frac{w_{\mathrm{low}}}{\bar{w}_{\mathrm{low}}}=W_{\mathrm{low}}$ is the relative fitness of an individual experiencing relatedness *r*_low_ and that $\frac{w_{\mathrm{high}}}{\bar{w}_{\mathrm{high}}}=W_{\mathrm{high}}$ is the relative fitness of an individual experiencing relatedness *r*_high_. Accordingly, incorporating equations (A2-3) into equation (A1) in the context of the illustrative model present in main text results in

$W=q\frac{\left( \frac{1-x_{\mathrm{low}}}{1-y_{\mathrm{low}}} \right)^{\beta}y_{\mathrm{low}}}{\bar{z}}+\left( 1-q \right)\frac{\left( \frac{{1-x}_{h\mathrm{ig}h}}{{1-y}_{h\mathrm{ig}h}} \right)^{\beta}y_{h\mathrm{ig}h}}{\bar{z}},$ (A4)

where: *x*_i_ is the investment into cooperation by the focal individual experiencing relatedness *r*_i_; *y*_i_ is the investment into cooperation by the individuals experiencing relatedness *r*_i_ in the focal patch; *z*_i_ is the average investment into cooperation by the individuals in the population

experiencing relatedness *r*_i_; i = {low, high}; and $\bar{z}=q\left( z_{\mathrm{low}} \right)+(1-q)(z_{\mathrm{high}})$ is the average investment into cooperation in the population.

Following Taylor & Frank (1996) approach, we may write d*W*_i_/d*g*_i_ = (∂*W*_i_/∂*x*_i_)(d*x*_i_/d*G*_i_)(d*G*_i_/d*g*_i_) + (∂*W*_i_/∂*y*_i_)(d*y*_i_/d*G*_i_*’*)(d*G*_i_*’*/d*g*_i_) where: *G*_i_ is the focal individual’s breeding value; *G*_i_*’* is the average breeding value of the individuals in the focal patch; d*x*_i_/d*G*_i_ = d*y*_i_/d*G*_i_*’* = *γ*_i_ is the mapping between genotype and phenotype; d*G*_i_/d*g*_i_ = *p*_i_ is the consanguinity of the gene in the focal individual to the individual herself; and d*G*_i_*’*/d*g*_i_ = *p*_i_*’* is the consanguinity of the gene in the focal individual with a randomly-chosen individual on her patch. We divide all terms of the right side of the equation by *p*_i_ to get the kin-selection coefficient of relatedness (Bulmer 1994):

$q\left( \frac{{\partial W}_{\mathrm{low}}}{{\partial x}_{\mathrm{low}}}\gamma_{\mathrm{low}}+\frac{{\partial W}_{\mathrm{low}}}{{\partial y}_{\mathrm{low}}}{\gamma_{\mathrm{low}}r}_{\mathrm{low}} \right)+\left( 1-q \right)\left( \frac{{\partial W}_{\mathrm{high}}}{{\partial x}_{\mathrm{high}}}\gamma_{\mathrm{high}}+\frac{{\partial W}_{\mathrm{high}}}{{\partial y}_{\mathrm{high}}}\gamma_{\mathrm{high}}r_{\mathrm{high}} \right)>0$ (A5)

We assume now that individuals are incapable of kin discrimination (γ_low_ = γ_high_ = 1). Accordingly, evaluating the derivatives at *x*_i_ = *y*_i_ = *z*_i_ = *z*, we obtain the following marginal fitness equation for the model in the absence of kin discrimination:

$q\left( -\frac{z^{*}\beta}{{(1-z}^{*}) \bar{w}_{U}}+r_{\mathrm{low}}\frac{(1-z^{*})+z^{*}\beta}{(1-z^{*}) \bar{w}_{U}} \right)+\left( 1-q \right)\left( -\frac{z^{*}\beta}{{(1-z}^{*}) \bar{w}_{U}}+r_{\mathrm{high}}\frac{(1-z^{*})+z^{*}\beta}{(1-z^{*}) \bar{w}_{U}} \right)=0$, (A6)

where $\bar{w}_{U}$ = *q* (*z^*^*) + (1 – *q*)(*z^*^*) and *z^*^* is the optimal investment into cooperation. Solving equation (A6) for *z^*^* returns the optimal of investment into cooperation that individuals should make in the absence of kin discrimination:

$z^{*}=\frac{r}{r+\beta(1-r)}$, (A7)

where $r$ = *q* *r*_low_ + (1 – *q*) *r*_high_ is the average relatedness in the population, which is the same as equation (1) of the main text.

We assume now that individuals are capable of kin discrimination. We first focus on the individuals experiencing relatedness *r*_low_ (γ_low_ = 1; γ_high_ = 0). Accordingly, evaluating the derivatives at *x*_low_ = *y*_low_ = *z*_low_, we can obtain the following marginal fitness equation

$q\left( -\frac{{z_{\mathrm{low}}}^{*}\beta}{{{(1-z}_{\mathrm{low}}}^{*}) \bar{w}_{D}}+r_{\mathrm{low}}\frac{{{(1-z}_{\mathrm{low}}}^{*})+{z_{\mathrm{low}}}^{*}\beta}{(1-{z_{\mathrm{low}}}^{*}) \bar{w}_{D}} \right)=0$, (A8)

where: $\bar{w}_{D}$ = *q* ${z_{\mathrm{low}}}^{*}$+ (1 – *q*) ${z_{\mathrm{high}}}^{*}$; ${z_{\mathrm{low}}}^{*}$ is the optimal level of resources that the individuals should take for themselves when experiencing relatedness *r*_low_; and ${z_{\mathrm{high}}}^{*}$ is the optimal level of resources that the individuals should take for themselves when experiencing relatedness *r*_high_.

Now we focus on individuals experiencing relatedness *r*_high_ (γ_low_ = 0; γ_high_ = 1). Accordingly, evaluating the derivatives at *x*_high_ = *y*_high_ = *z*_high_, we can obtain the following marginal fitness equation:

$(1-q)\left( -\frac{{z_{\mathrm{high}}}^{*}\beta}{{{(1-z}_{\mathrm{high}}}^{*}) \bar{w}_{D}}+r_{\mathrm{high}}\frac{{{(1-z}_{\mathrm{high}}}^{*})+{z_{\mathrm{high}}}^{*}\beta}{(1-{z_{\mathrm{high}}}^{*}) \bar{w}_{D}} \right)=0$. (A9)

Finally, we obtain the optimal level of resources taken by the individuals in the population experiencing relatedness *r*_low_ and the optimal level of resources taken by the individuals in the population experiencing relatedness *r*_high_ by solving the system of equations (A8-A9) for *z*_low_*^*^* and *z*_high_*^*^*:

${z_{\mathrm{low}}}^{*}=\frac{r_{\mathrm{low}}}{r_{\mathrm{low}}+\beta(1-r_{\mathrm{low}})}$; (A10)

${z_{\mathrm{high}}}^{*}=\frac{r_{\mathrm{high}}}{r_{\mathrm{high}}+\beta(1-r_{\mathrm{high}})}$, (A11)

which are the same as equation (3) and equation (4), respectively, of the main text.

**References**

1. Taylor PD, Frank SA. 1996 How to make a kin selection model. *J Theor Biol* **180**, 27–37.
2. Bulmer MG. 1994 *Theoretical evolutionary ecology.* Sunderland, MA: Sinauer Associates.
